# Comparison of Recombinant MVA Selection Methods Based on F13L, D4R and K1L Genes

**DOI:** 10.3390/v14030528

**Published:** 2022-03-04

**Authors:** Irina V. Antoshkina, Dina V. Glazkova, Felix A. Urusov, Elena V. Bogoslovskaya, German A. Shipulin

**Affiliations:** 1 Federal State Budgetary Institution «Centre for Strategic Planning and Management of Biomedical Health Risks» of the Federal Medical Biological Agency, 119121 Moscow, Russia; georgin2702@gmail.com (D.V.G.); flanger.fx@mail.ru (F.A.U.); lenabo2@mail.ru (E.V.B.); shipgerman@gmail.com (G.A.S.); 2 Izmerov Research Institute of Occupational Health, 105275 Moscow, Russia

**Keywords:** MVA, vaccinia virus, recombinant MVA, host-range selection

## Abstract

Modified vaccinia Ankara (MVA) is a promising vaccine vector due to its highly attenuated phenotype and good immunogenicity. However, obtaining a new recombinant MVA remains a tedious and laborious procedure involving many rounds of plaque purification. Recombinant MVA generation can be greatly improved and facilitated by different selection techniques. Here, we describe a comparison between techniques based on K1L, F13L and D4R genes.

## 1. Introduction

For many years, the modified virus Ankara (MVA) has been widely used as a viral vector for the development of numerous vaccines that are presently at different stages of clinical and preclinical trials. Vaccine candidates against tuberculosis, malaria, flu, HIV and MERS expressing transgenic antigens have been developed [1].

Some features of the virus have contributed to that popularity of the vector. MVA was obtained in Germany as a result of multiple passages of vaccinia virus (VACV) Ankara in chicken embryo fibroblasts, which led to the loss of some part of the genome and the ability to replicate in mammalian cells, including human cells (except for the cell line BHK-21) [2].

Such an attenuated phenotype is beneficial from a safety perspective, which was proven on more than 120,000 people during vaccination against smallpox [3]. At the same time, MVA as a viral vector provides a high level of expression of the delivered antigen, and as a result, it exerts a potent immunogenic effect, thus provoking humoral and cellular responses [4]. Along with the high capacity of a vector for recombinant DNA and the simplicity of handling vectors in laboratory conditions, these features make MVA a promising viral vector for the development of effective vaccines.

Recombinant strains of MVA are obtained by homologous recombination between the genome of the virus and the vector plasmid that carries the target gene. Recombination occurs in host cells during the replication of the virus. However, the rate of homologous recombination is very low, and the obtained virus progeny primarily consists of a parental virus (99.9%) [5]; therefore, it is necessary to enrich and isolate the recombinant virus after recombination.

A traditional method of recombinant virus purification is the isolation of individual virus plaques. This method contains several stages and is labor- and time-consuming, particularly because of the difficulty of visualizing and analyzing plaques. Thus, other methods were developed that simplified this process. For example, some of them were based on co-expression of genes allowing resistance to some substance, such as mycophenolic acid (xanthine transferase gene-based selection (*gpt*) from *E. coli* ) [6] or puromycin [7]. Another method of selection involved the application of different markers that stain the plaques. The application of β-galactosidase and chromogenic substrate (X-gal) stains plaques blue [8,9], and the application of fluorescent proteins enables the selection of plaques by fluorescence [10].

However, even when selective markers are used, the obtained plaques contain a significant share of the initial virus, and the isolation of pure recombinants can be time-consuming. Attempts have been made to accelerate the procedure of plaque selection; in particular, additional approaches to the acceleration of recombinant plaque selection using a semiquantitative PCR test were proposed [11]. The proposed approach allowed us to select plaques with the maximal content of recombinant virus, thus accelerating the procedure of selection of the recombinant MVA viruses. However, the laborious character of the process associated with the regular selection of plaques limits the application of this approach.

The attempts to simplify the procedure of recombinant virus purification led to the development of selection methods based on altering of MVA replication process. For this, some of the Poxvirus genes that rescue MVA growth in certain host cells are inserted into the MVA genome along with the target gene.

For example, the insertion into the MVA of a Poxvirus K1L gene, which is lost during attenuation, allows a recombinant virus to replicate in rabbit kidney cell line RK-13. During passaging of a viral mixture of a wild-type and a recombinant MVA only the latter will replicate in this cell line, which provides the selection of rMVA [12].

In addition, approaches based on the application of MVA with weakened growth obtained due to the deletion of certain genes have been developed. One such gene is F13L. Its deletion slows the rate of virus reproduction and results in a small plaques phenotype [5]. A deletion of another gene, D4R, leads to a complete loss of replication ability in cells permissive for MVA [13]. The restoration of a deleted gene in the MVA genome during recombination leads to the reacquisition of the growth rate of a wild strain of the virus. During further passages of the mixture of the initial and recombinant viruses, the growth of the parental strain with a deletion will be suppressed.

The aim of the study was to test and compare the above-mentioned selection methods.

## 2. Materials and Methods

### 2.1. Cells and Viruses

Baby hamster kidney cells BHK-21 (ATCC ^®^ CCL-10) and BHK-21-D4R and human embryonic kidney HEK293FT (Invitrogen, Carlsbad, CA, USA) cells were grown in DMEM (Gibco, Waltham, MA, USA) supplemented with 4.5 g/L D-glucose, 10% fetal bovine serum (FBS) (Gibco, Waltham, MA, USA) and 0.01 M HEPES (Gibco, Waltham, MA, USA). Rabbit kidney cells RK-13 (ATCC^®^ CCL-37) were grown in MEM (Gibco, Waltham, MA, USA) supplemented with 10% FBS (Gibco, Waltham, MA, USA), 0.01 M HEPES (Gibco, Waltham, MA, USA) and 0.1 mM nonessential amino acids (Gibco, Waltham, MA, USA). The cells were maintained in a 5% CO_2_ humidified atmosphere at 37 °C.

MVA and VACV strain WR were obtained from ATCC (VR-1508 and VR-2035, respectively).

### 2.2. Plasmids

**Figure 1:**
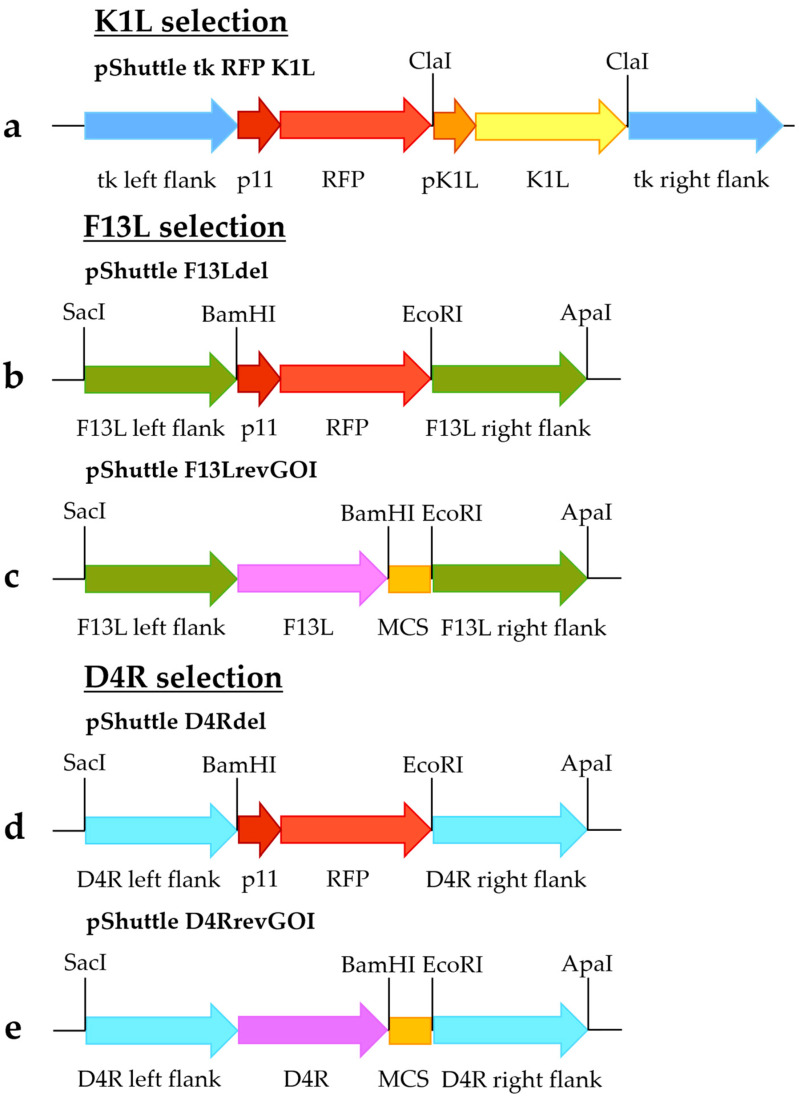
Shuttle vectors for selection. (**a**) pShuttle tk RFP K1L; (**b**) pShuttle F13Ldel; (**c**) pShuttle F13LrevGOI; (**d**) pShuttle D4Rdel; and (**e**) pShuttle D4RrevGOI. MCS—multiple cloning site.

The shuttle vector pShuttle tk RFP K1L (Figure 1a) was constructed as previously described [12]. The sequence containing K1L controlled by its promoter was amplified using primers 1 and 2 specified in Table 1 and VACV strain Western Reserve as a template. The PCR product was digested by ClaI and inserted into the corresponding site in the pShuttle tk RFP plasmid, which contained the red fluorescent protein (RFP) gene flanked by the MVA thymidine kinase gene flanking sequences. The insert orientation was checked by sequencing. Generation of the pShuttle tk RFP vector was previously described [11].

To obtain pShuttle F13Ldel (Figure 1b) and pShuttle D4Rdel (Figure 1d) shuttle vectors, flanking sequences of the F13L and D4R genes were amplified. Fragments containing left recombination arms of F13L and D4R (using primers 3 and 4 for F13L [5] and primers 13 and 14 for D4R [13], Table 1) and right recombination arms (primers 5 and 6 for F13L and primers 15 and 16 for D4R, Table 1) were amplified using the MVA genome as a template. In addition, a fragment containing RFP controlled by the tk promoter was amplified using primers 7 and 8 and plasmid pShuttle tk RFP as a template. The fragments were digested with restriction enzymes specified in Table 1 and Figure 1 and cloned between SacI/ApaI restriction sites of the pGEM-T Easy vector (Promega, Madison, WI, USA) (Figure 1b,d).

Shuttle vectors pShuttle F13LrevGOI (Figure 1c) and pShuttle D4RrevGOI (Figure 1e) were constructed by replacing the left flanking sequences in the pShuttle F13Ldel and pShuttle D4Rdel shuttle vectors with fragments containing the left F13L/D4R flanking sequences along with complete F13L/D4R genes, respectively. These fragments were amplified using the MVA genome as a template, with primers 9 and 10 for the F13L fragment and primers 19 and 20 for the D4R fragment (specified in Table 1) and digested by SacI/BamHI sites. In addition, a fragment containing a multiple cloning site was obtained by restriction of the plasmid pShuttle tk RFP by BamHI/EcoRI sites. The fragments were cloned into SacI/EcoRI-digested shuttle vectors pShuttle F13Ldel (Figure 1b) or pShuttle D4Rdel (Figure 1d).

To generate the lentiviral vector pLV D4R, a fragment containing D4R controlled by its promoter was amplified using primers 11 and 12 and the MVA genome as a template and cloned into the XbaI/BamHI-digested vector pG with EGFP as a marker gene (previously described in [14]).

### 2.3. Generation of Recombinant MVA

Recombinant MVA viruses (rMVA) were generated by homologous recombination [15]. A confluent monolayer of BHK-21 cells grown in a 6-well plate was infected with MVA (0.05 PFU/cell). Ninety minutes after infection, the medium was replaced with 2 mL of fresh DMEM supplemented with 2% FBS, and the cells were transfected with 3 µg of plasmid DNA using FuGENE HD Transfection Reagent (Promega, Madison, WI, USA). After 48 h of incubation, the cells were detached and lysed with three cycles of freeze/thawing. For subsequent infection, the virus stocks were sonicated.

### 2.4. RK-13 Cells Infection and Plaques Isolation

A confluent monolayer of RK-13 cells grown in a 6-well plate was infected in a total volume of 2 mL per well with serial dilutions of viral lysates obtained from homologous recombination or separate plaques and incubated for 72 h. Separate plaques were picked with a pipette directly from the cell monolayer, transferred into 500 µL of MEM and lysed with three cycles of freeze/thawing. For subsequent infection, the virus stocks were sonicated.

### 2.5. BHK-21 Cells Infection (Virus Serial Passaging)

A confluent monolayer of BHK-21 cells grown in a 6-well plate was infected with 100 µL of the virus stock after recombination (total volume was 2 mL of medium per well). The cells were incubated for 48 h, detached and lysed with three cycles of freeze/thawing. The obtained stock was used for the next passage (50 µL per well of a 6-well plate) [13,16].

### 2.6. Purification of MVA by Plaque Isolation

A confluent monolayer of BHK-21 or BHK-21-D4R cells grown in a 6-well plate was infected with serial dilutions of viral lysates obtained from homologous recombination or separate plaques. Ninety minutes after infection, the medium was replaced with 2 mL of fresh DMEM supplemented with 2% FBS and 1% low-melting point agarose. Forty-eight hours after infection, the fluorescent plaques were picked through agarose overlay.

### 2.7. Cloning by the Limiting Dilution Method

A confluent monolayer of BHK-21 or BHK-21-D4R or RK-13 cells grown in a 48-well plate was infected with serial dilutions of viral lysates and incubated for 72 h. Then, cells in wells that contained single plaques of the respective phenotype were detached and used as a crude virus stock.

### 2.8. Determination of Infectious Titers

A virus titer was determined using BHK-21/BHK-21-D4R cell lines and the TCID50 method previously described [15].

### 2.9. Generation of a BHK-21-Based Cell Line Expressing the D4R Gene

The D4R gene was introduced into BHK-21 cells via lentiviral vector transduction.

Lentiviral vector particles were obtained by a standard method of transient transfection (previously described in [17]) of HEK293FT cells with packaging plasmids Lenti-X HT Packaging Mix (Clontech, Mountain View, CA, USA) and vector plasmid pLV D4R. After 72 h, the supernatant containing vector particles was collected and filtered through 0.45 μm filters.

For target cell line generation, BHK-21 cells (10^5^ per well in a 24-well plate) were infected with 200 µL of viral supernatant with the addition of 5 µg/mL polybrene (Sigma Aldrich, Burlington, MA, USA). On the next day, the medium was replaced with a fresh medium. Furthermore, the cells were seeded in a 96-well plate at a density of one cell per well, and EGFP-positive cells were selected. The selected EGFP+ clones were grown into separate cell lines.

### 2.10. Isolation of Virus DNA and PCR Performance

Viral DNA was isolated with the isopropanol precipitation method using the commercial “AmpliTest RIBO-prep” kit (Centre for Strategic Planning of FMBA of Russia, Moscow, Russia) according to the manufacturer’s recommendations.

For each selection system, we selected primers that anneal adjacent to the shuttle vector insertion sites in the MVA genome (tk locus, F13L locus and D4R locus). During amplification, fragments corresponding to rMVA and a wild-type virus are obtained that differ by length. All primers and amplification programs are listed in Table 2. The products of amplification were analyzed with gel electrophoresis. By the presence of products, the presence of the parent and target viruses in the viral mixture was determined.

## 3. Results

### 3.1. K1L-Based Selection

**Figure 2:**
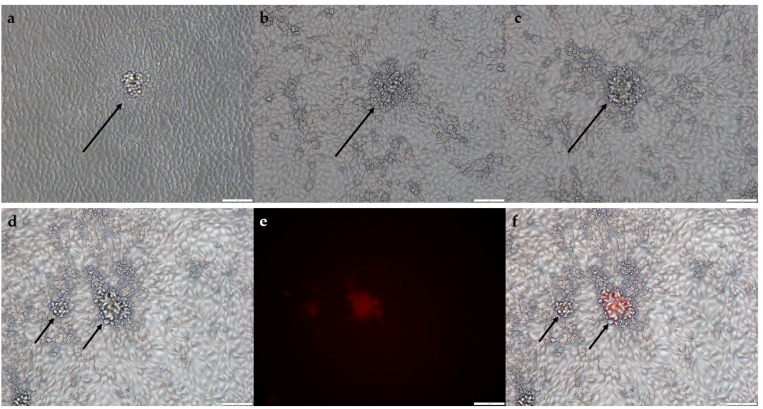
Microscopy of MVA—K1L-infected foci on RK-13 cell line. Viral plaques appear as cell aggregates above cell monolayer: (**a**–**c**) Bright-field microscopy of three different plaques; (**d**–**f**) Example of fluorescent plaque in bright-field (**d**) fluorescence microscopy (**e**), and merged image (**f**).

VACV contains the K1L gene that was lost in MVA. In the absence of the K1L gene, infection of RK-13 cells with the MVA virus leads to early blocking of viral replication. Reinsertion of the K1L gene into MVA as a part of the shuttle vector restores the capability of a recombinant virus to replicate in RK-13 cells. During the infection of these cells with a mix of parental and recombinant MVA, the plaques are formed only by rMVA that have the gene K1L in its genome. As parental virus growth is suppressed, the recombinant virus will be enriched with each round of plaque purification. In addition, the plaques appear as cell aggregates above the monolayer surface, which simplifies their selection because there is no need to use an agar medium during selection (Figure 2a–c) [12].

**Figure 3:**
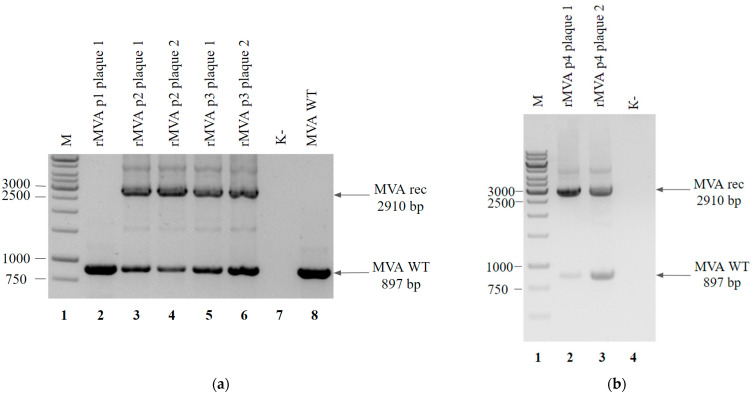

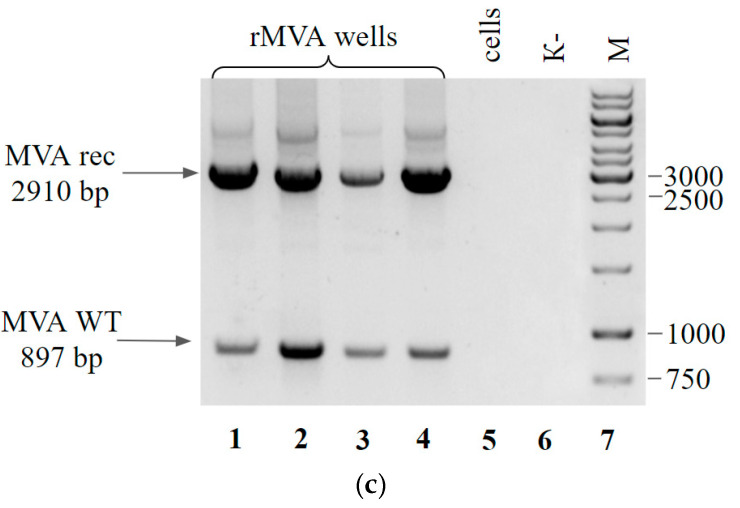
PCR analysis of the rMVA-K1L viral DNA from 1–4 selection rounds (**a,b**) and viral clones isolated by limiting dilution method using RK-13 cells (**c**): (**a**) Genomic DNA of virus plaques from passage 1 (lane 2), passage 2 (lanes 3 and 4), passage 3 (lanes 5 and 6) and wild-type MVA (MVA WT, lane 8) served as a template DNA. Negative control was run without any template (lane 7). Molecular weights were determined in comparison to the 1-kb ladder (lane 1); (**b**) Genomic DNA of virus plaques from passage 4 (lanes 2 and 3) served as a template DNA. Negative control was run without any template (lane 4). Molecular weights were determined in comparison to the 1-kb ladder (lane 1); (**c**) Cells from the wells that contained single plaques (lanes 1–4) and no plaques (lane 5) served as a template DNA. Negative control was run without any template (lane 6). Molecular weights were determined in comparison to the 1-kb ladder (lane 7).

To evaluate the effectiveness and convenience of this selection method, we designed a shuttle vector pShuttle tk RFP K1L that contained a marker gene of the red fluorescent protein (RFP), K1L gene, and homology arms to the thymidine kinase MVA locus (Figure 1a). To obtain a recombinant virus, a shuttle vector was transfected into BHK-21 cells infected with MVA. The obtained recombinant mixture was used to infect the RK-13 cell line. We selected fluorescent plaques (Figure 2c–e), and the obtained samples were used to infect the cells in the next round. The plaques selected in each round (about 20 plaques per round) were PCR tested to identify the presence of a wild-type virus and a recombinant virus (Figure 3a,b). For the next round we used the two plaques that presented a significant amount of the recombinant MVA (according to the PCR results). After the 4th round, separate viral clones were isolated by the limiting dilution method on RK-13 cells, and PCR testing was performed (Figure 3c).

We did not obtain a pure clone of rMVA-K1L in the three separate experiments starting from recombination. As shown in Figure 3a–c (results are presented for one of the three experiments), in all the analyzed clones, apart from the recombinant one, residual traces of a wild-type virus were present even when the limiting dilution method was used.

### 3.2. F13L-Based Selection

**Figure 4:**
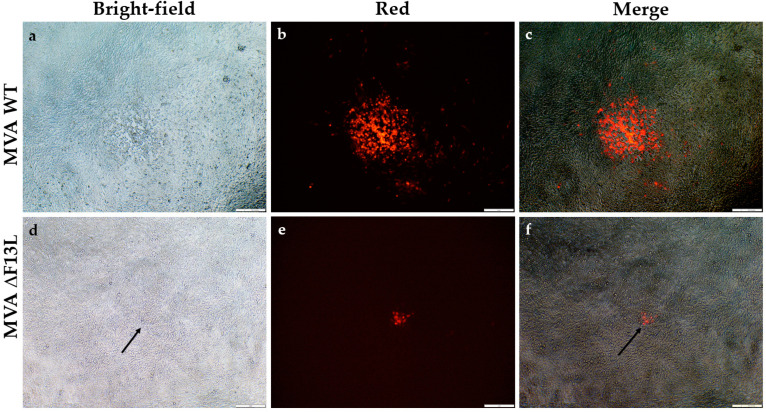
Microscopy of MVA WT (**a**–**c**) and MVA ΔF13L (**d**–**f**) foci on BHK-21 cell line: (**a**,**d**) Bright-field microscopy of MVA WT (**a**) and MVA ΔF13L (**d**); (**b**,**e**) Fluorescence microscopy of MVA WT (**b**) and MVA ΔF13L (**e**); (**c**,**f**) Merged images of MVA WT (**c**) and MVA ΔF13L (**f**).

This selection is based on obtaining the strain MVA ΔF13L that does not contain the native gene F13L [16] and further reinsertion of this gene in recombinant viruses along with the gene of interest. MVA ΔF13L cannot be effectively spread from cell to cell, which leads to a decreased rate of replication and a small plaque phenotype (Figure 4). The selection of recombinants that had the F13L gene restored is based on a significantly faster replication of recombinant clones in comparison to the MVA ΔF13L parent virus. In addition, the formation of large easily detectible nonfluorescent plaques by recombinant clones (unlike the initial MVA ΔF13L) facilitates the application of the limiting dilution method.

**Figure 5:**
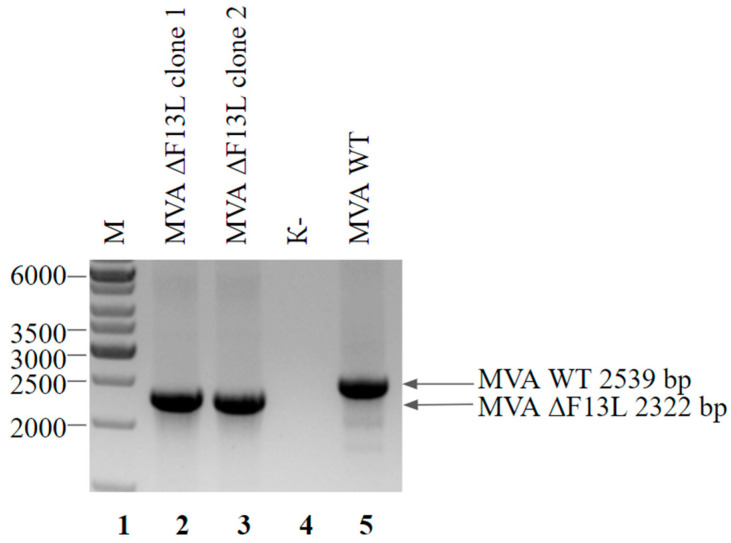
PCR analysis of the MVA ΔF13L viral DNA. Genomic DNA of MVA ΔF13L from clone 1 (lane 2), clone 2 (lane 3) and wild-type MVA (lane 5) served as a template DNA. Negative control was run without any template (lane 4). Molecular weights were determined in comparison to the 1-kb ladder (lane 1).

The first step in F13L-based selection is obtaining the virus MVA ΔF13L with a deletion of this gene in the genome. To obtain MVA ΔF13L, we designed a shuttle vector pShuttle F13Ldel, which contained the RFP gene flanked by homology arms to locus F13L. During recombination with a shuttle vector, the gene F13L in the MVA genome was replaced with a marker gene RFP. Pure MVA ΔF13L virus was isolated by several rounds of plaque purification. PCR analysis of the obtained clones is presented in Figure 5.

To obtain recombinant MVA with restored growth properties, we designed the shuttle vector pShuttle F13LrevGOI that contained arms of homology to the locus F13L and a native gene F13L. Recombination between pShuttle F13LrevGOI and MVA ΔF13L genome mediates the replacement of the RFP gene with the F13L gene, restoring the F13L gene into its original locus. The lack of RFP fluorescence allows the differentiation of a recombinant virus from parent MVA ΔF13L. Moreover, after obtaining a pure recombinant rMVA F13Lrev, additional deletion of the RFP sequence is not required.

**Figure 6:**
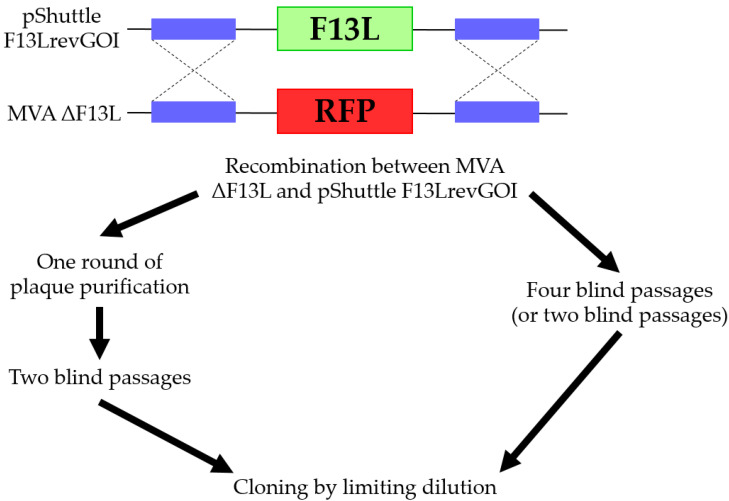
Experimental scheme for isolating MVA recombinants using F13L.

To perform recombination, pShuttle F13LrevGOI was transfected into BHK-21 cells preliminarily infected with MVA ΔF13L. Further, we purified the recombinant MVA using two different protocols proposed by [5] (Figure 6). The first protocol consisted of four consecutive blind virus passages in BHK-21 cells. The second protocol included one round of plaque purification and then two blind passages of the isolated plaques. After the last step of both protocols, individual viral clones were isolated by the limiting dilution method. We selected wells that contained single nonfluorescent large plaques and analyzed them by PCR.

**Figure 7:**
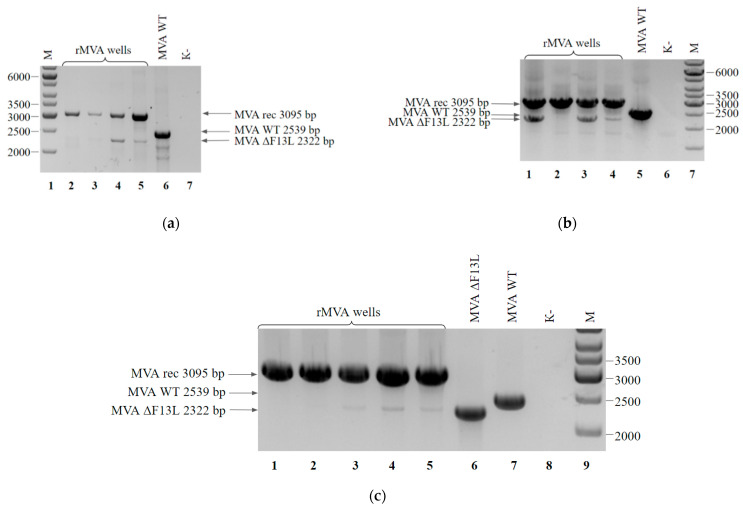
PCR analysis of rMVA-F13Lrev viral DNA: (**a**) Clones after four blind passages and isolation by limiting dilution method. Cells from the wells contained single plaques (lanes 2–5) and wild-type MVA (lane 6) served as a template DNA. Negative control was run without any template (lane 7). Molecular weights were determined in comparison to the 1-kb ladder (lane 1); (**b**) Clones after one round of plaque isolation, subsequent two blind passages of selected plaques and isolation by limiting dilution method. Cells from the wells contained single plaques (lanes 1–4) and wild-type MVA (lane 5) served as a template DNA. Negative control was run without any template (lane 6). Molecular weights were determined in comparison to the 1-kb ladder (lane 7); (**c**) Clones after two blind passages and isolation by limiting dilution method. Cells from the wells contained single plaques (lanes 1–5), MVA ΔF13L (lane 6) and wild-type MVA (lane 7) served as a template DNA. Negative control was run without any template (lane 8). Molecular weights were determined in comparison to the 1-kb ladder (lane 9).

As shown in Figure 7a,b, pure clones of the rMVA were obtained by each of the tested methods. Nearly half of the selected clones contained a recombinant without traces of the parent virus. The second method that included the plaque isolation took two days fewer than the first one (12 and 14 days, including recombination, respectively). However, it should be noted that the first method is less laborious and simpler because it only requires blind passaging.

We also tested the possibility of accelerating the rMVA purification using the faster purification protocol. We tried to reduce the number of passages for a viral mixture in the first protocol from four to two. After that, separate clones were isolated by the limiting dilution method. There were small fluorescent plaques of the parent virus in numerous wells, although we managed to select 5 wells with target single nonfluorescent large plaques from 16 wells. The obtained clones were analyzed by PCR. As a result, two clones of pure recombinant were selected (Figure 7c). Thus, it was possible to reduce the period of recombinant isolation to nine days.

### 3.3. D4R-Based Selection

**Figure 8:**
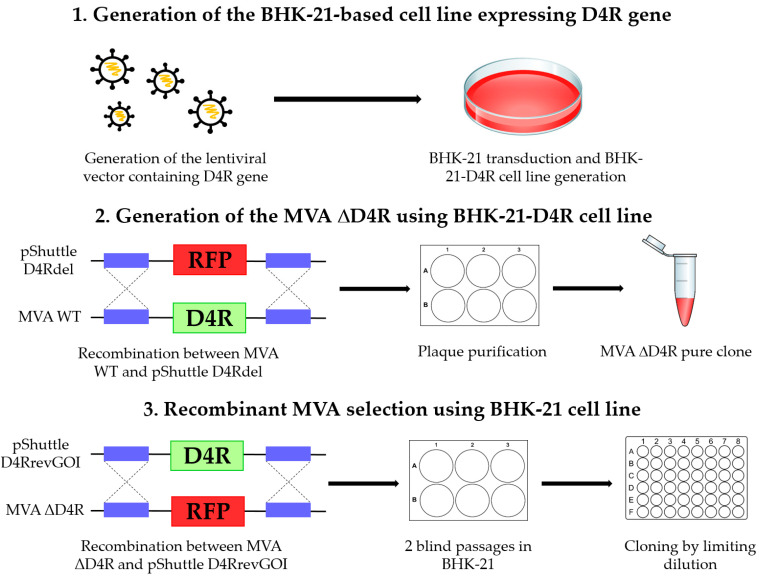
Experimental scheme for isolating MVA recombinants using D4R.

Method of selection based on the vaccinia virus D4R gene was first described by [13]. D4R encodes the protein uracil DNA glycosylase that is necessary for virus replication. Mutant MVA ΔD4R with a deletion of this gene is not capable of replicating in ВНК-21 cells. Thus, growth of the knock-out virus requires an engineered cell line that complements the function of the deleted gene by expressing the D4R gene. MVA ΔD4R, which is capable of growing only on these cells, is used as a parental virus. The selection of rMVA is based on the reinsertion of the D4R gene, as in the case with F13L, and enrichment of virus recombinants by serial passages in BHK-21 cells that could provide growth only to the revertant virus (Figure 8).

To obtain a BHK-21-D4R cell line that expresses D4R, we designed a lentivirus vector LV D4R containing the D4R gene controlled by its promoter and EGFP controlled by the PGK promoter. The vector was transduced into BHK-21 cells, and EGFP-positive clones were selected and grown into cell lines.

**Figure 9:**
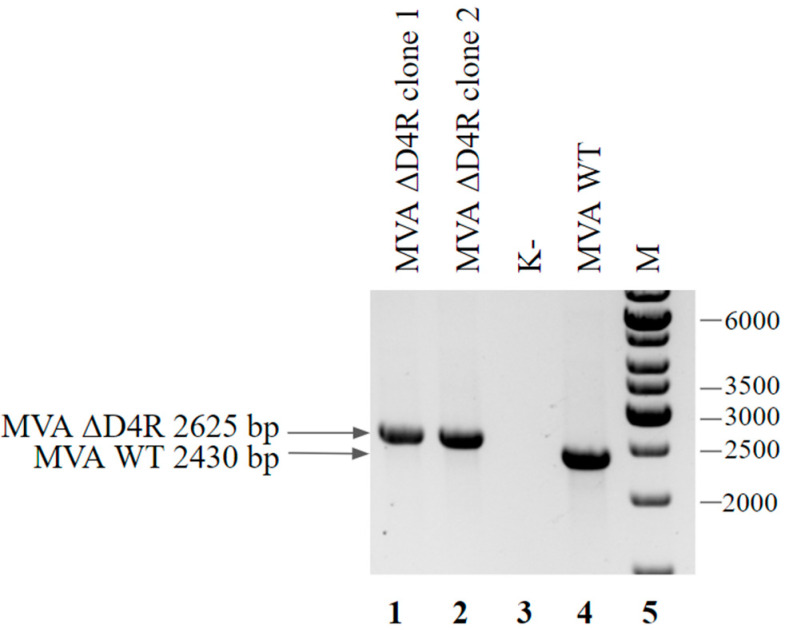
PCR analysis of MVA ΔD4R viral DNA. Genomic DNA of MVA ΔD4R from clone 1 (lane 1), clone 2 (lane 2) and wild-type MVA (lane 4) served as a template DNA. Negative control was run without any template (lane 3). Molecular weights were determined in comparison to the 1-kb ladder (lane 5).

To obtain the MVA virus with D4R deletion, we designed a shuttle vector pShuttle D4Rdel, which contained the RFP gene flanked by homology arms to locus D4R. For recombination, a shuttle vector was transduced into BHK-21-D4R cells preliminarily infected with MVA. Pure virus MVA ΔD4R was obtained by plaque purification. The results of PCR analysis of the obtained clones are presented in Figure 9.

To obtain MVA recombinants with restored replication, we designed the shuttle vector pShuttle D4RrevGOI containing flanking sequences of D4R along with the complete D4R gene. Recombination between the pShuttle D4RrevGOI and MVA ΔD4R genome mediates the replacement of the RFP gene with the D4R gene, restoring the D4R gene into its original locus. Therefore, after obtaining pure recombinant MVA, the additional removal of a fluorescent marker is not required.

**Figure 10:**
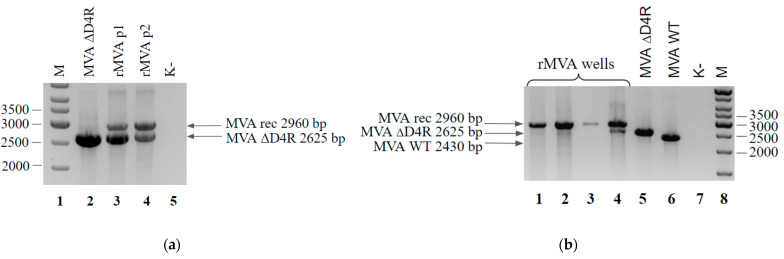
(**a**) PCR analysis of rMVA-D4Rrev viral DNA from the two blind passages on the BHK-21 cells. Genomic DNA of the MVA ΔD4R (lane 2), first passage (lane 3) and second passage (lane 4) served as a template DNA. Negative control was run without any template (lane 5). Molecular weights were determined in comparison to the 1-kb ladder (lane 1); (**b**) PCR analysis of rMVA-D4Rrev viral DNA of the clones isolated by limiting dilution method after two blind passages. Cells from the wells contained single plaques (lanes 1–4), MVA ΔD4R (lane 5), and wild-type MVA (lane 6) served as a template DNA. Negative control was run without any template (lane 7). Molecular weights were determined in comparison to the 1-kb ladder (lane 8).

To perform recombination, pShuttle D4RrevGOI was transfected into BHK-21-D4R cells preliminarily infected with MVA ΔD4R. After recombination, the viral mixture underwent two blind passages in BHK-21 cells. The authors of this selection method noted that after two passages the viral mixture contained only a recombinant. We analyzed the PCR results of the viral mixture after the 2nd passage and revealed that there were residual traces of the parent MVA ΔD4R virus (Figure 10a). Thus, additional isolation of single clones by the limiting dilution method was performed. Out of 10 analyzed clones, only one contained traces of the parent virus (Figure 10b). In total, this process took nine days (including recombination).

## 4. Discussion

To obtain an MVA-based vaccine, it is necessary to integrate the sequence encoding antigens into the viral genome. It is carried out by homologous recombination between the initial virus and shuttle vector with further isolation of the target recombinant MVA by plaque purification. This process is rather laborious and inefficient. Previously, we tried to optimize a conventional method of rMVA selection by adding a real-time PCR test of single plaques [11], which allowed for the acceleration of recombinant virus selection. However, the process remained laborious and did not allow us to obtain numerous recombinants simultaneously.

Several methods of recombinant virus purification based on the enrichment of rMVA during replication have been developed to simplify the process. However, until now these methods have not been compared directly. Thus, the aim of the present study was to compare the efficiency and usability of some of them.

The first tested method of rMVA isolation was based on the host range gene, K1L, as the selective growth marker in rabbit kidney RK-13 cells. We showed that such a method of rMVA selection did not perform well. The publications mentioned various periods required for isolation of pure recombinant virus (from 2 to 5 selective passages in RK-13) [18,19]. In our turn, we did not manage to obtain pure recombinant after four rounds of plaque selection, which is a more effective selection than just blind passaging. Even after the application of additional limiting dilution cloning, each of the tested clones had residual traces of wild-type MVA. The problem of rMVA selection using RK-13 was also described in the work by [19], who suggested that the recombinant virus (K1L^+^) allows the rescue of the wild-type virus (K1L^−^) in the same cell.

Further, we tested two similar systems of selection based on the deletion of F13L or D4R from the MVA genome and further reinsertion of these genes, which are necessary for successful replication. The main difference between MVA ΔF13L and MVA ΔD4R was in the rate of replication. MVA ΔF13L replication is dramatically reduced in the BHK-21 cell culture compared to wild-type MVA, while MVA without essential D4R gene could not replicate in these cells at all. Since MVA ΔD4R was not capable of replicating in cells, for its propagating it was required to additionally generate a cell line expressing the D4R to complement the absent gene function.

We successfully obtained pure recombinant viruses without admixtures of the parental viruses using both systems. Although D4R-based selection was more effective. Nine out of ten (90%) viral clones, isolated by the limiting dilution method, did not contain the admixtures of the initial MVA ΔD4R after two blind passages. On the other hand, for F13L selection approximately 12% and 50% of such samples were obtained after two and four blind passages correspondingly.

Both systems were simple and convenient in practice. First, there is no need for the application of antibiotics, selective agents, agar medium, etc. Second, a recombinant virus can be obtained after two to four blind passages without laborious plaque isolation and analysis of plaques at each stage. Third, since passaging is not time-consuming, several recombinant viruses can be obtained simultaneously. Fourth, the advantage of this method compared to the conventional method is that it does not require additional marker genes to select a recombinant sequence and thus does not need further removal of these sequences.

The disadvantage of both systems is that the insertion of the target gene is possible only in the initial locus, where the genes F13L and D4R are located. Furthermore, this type of selection can be used for the integration of only one insert because the reinsertion of F13L/D4R along with the target gene restores the native growth properties of the virus.

When it is necessary to obtain rMVA with the additional insert in a different locus, the methods of rMVA selection should be combined. For example, F13L/D4R-based selection can be used for the first integration. For the second integration, it is possible to use an accelerated method of selection of single plaques using real-time PCR [11]. The application of this approach allowed us to obtain rMVA that had insertions in the F13L locus and 136/137 intergene area. In this case, we omitted the limiting dilution cloning during the first recombinant purification (F13L selection), which did not influence the further process of selection of double recombinants (unpublished data). As a result, double recombinants were obtained in 16 days.

Additionally, the approach based on the application of MVA with the deletion of both genes (MVA ΔF13L ΔD4R) is promising because it can reduce the time for isolation of double recombinants. However, the prospects of this approach have yet to be elucidated.

## Figures and Tables

**Table 1:** Primers used in this study for cloning.

Number	Primer Sequence (Restriction Site)
1	CAGCATCGATTGCGATAGCCATGTATCTACTAATCAG (ClaI)
2	GCAGATCGATGGAAATCTATCTTATATACAC (ClaI)
3	GGGGAGCTCGATAAAGTTTCGAAACAGCAAA (SacI)
4	CATTTTGGGATCCCAGGTACCGGTGCAA (BamHI)
5	GAGAGAATTCGGGTATCTAGCCACAGTA (EcoRI)
6	CACAGGGCCCCTCTAGATATGTATTTAA (ApaI)
7	CCCGGGATCCATCGATGAAGGACAGTTCTA (BamHI)
8	CCCGGAATTCTTATTAATTAAGTTTGTGCC (EcoRI)
9	GGGGAGCTCGATAAAGTTTCGAAACAGCAAA (SacI)
10	CCCGGATCCTTAAATTTTTAACGATTTACTGTGGCTAGATACCCAATCTCTCTCAAAT (BamHI)
11	GCAGGATCCAGGCGTTTGTATTCGCTTGG (BamHI)
12	CTAGATCCTTTAATAAATAAACCCTTGAGCC (BamHI)
13	CCCGGAGCTCTGTGAGCTACTGTAGGTG (SacI)
14	CCCGTCTAGATTATATCAAATTAGATACC (XbaI)
15	CCCGGAATTCGCTTTAGTGAAATTTTAAC (EcoRI)
16	CCCGGGGCCCACTATTGTTGTTCATATCCACG (ApaI)
17	AGAGTCTAGAATCGATGAAGGACAGTTCTATACATAG (XbaI)
18	AGAGGAATTCTTATTAATTAAGTTTGTGCCCCAGTTTG (EcoRI)
19	CCCGGAGCTCTGTGAGCTACTGTAGGTG (SacI)
20	CCCGGGATCCTTAATAAATAAACCCTTGAGCC (BamHI)

**Table 2:** List of primers and thermocycling conditions.

Selection Method	Primer	Sequence	PCR Product Size	Thermocycling Conditions
K1L	TKL new	GCTACCACCGCAATAGATCCT	897 bp for wild-type MVA; 2910 bp for rMVA-K1L	Initial denaturation at 95° for 5 min; followed by 40 cycles at 95° for 30 s, 55° for 30 s, 72° for 4 min; and a final extension at 72° for 5 min.
	TKR new	CTAATATACCGTGTCGCTGTAAC		
F13L	a49	CCTCAGTTTCAATATCTCCTTCCTG	2539 bp for wild-type MVA; 2322 bp for MVA ΔF13L; 3095 bp for rMVA F13Lrev	Initial denaturation at 95° for 5 min; followed by 40 cycles at 95° for 30 s, 52° for 30 s, 72° for 4 min; and a final extension at 72° for 5 min.
	a50	GTTTTTATATGTGTTTGATCTAACGAG		
D4R	a58	ACCTTCCAACTGTGGATACTCTG	2430 bp for wild-type MVA; 2625 bp for MVA ΔD4R; 2960 bp for rMVA D4Rrev	Initial denaturation at 95° for 5 min; followed by 40 cycles at 95° for 30 s, 52° for 30 s, 72° for 4 min; and a final extension at 72° for 5 min.
	a59	TCGAATGAAATAAACCCTGGT		

## Data Availability

The original data presented in the study can be seen on request to the corresponding authors.
